# Experimental study on uniaxial creep characteristics of sandstone with pre-peak unloading damage

**DOI:** 10.1038/s41598-022-20505-z

**Published:** 2022-09-28

**Authors:** Zhonghao Liang, Peizhi Ji, Yifan Zhang, Nan Qin, Qiming Huang, Zhuoqun Yu

**Affiliations:** 1 School of Mechanical and Electrical Engineering, Qingdao University of Science and Technology, Qingdao, 266061 China; 2 College of Safety and Environmental Engineering, Shandong University of Science and Technology, Qingdao, 266590 China; 3 School of Civil Engineering, Yantai University, Yantai, 264005 China

**Keywords:** Civil engineering, Petrology, Economic geology

## Abstract

Deep resource extraction has been affected by the complex geological environment of "three highs and one disturbance" for a long time. The surrounding rocks experience strong unloading stress disturbance during the underground resource extraction. The creep characteristics of the perimeter rocks are of great theoretical and practical value. Firstly, the triaxial pre-peak loading and unloading tests on the prepared samples are conducted on the intact rock samples using the TAW-200 rock mechanics test system. Then the rock samples with different degrees of pre-peak unloading damage were prepared under the condition of pre-peak yield. Finally, the uniaxial creep tests were carried out to study the uniaxial creep mechanical properties. The results show that the wave velocities of the damaged rock samples are reduced to different degrees compared with those of the intact rock samples, the creep of the pre-peak unloading damaged rock has a time-dependent damage effect, and the study results are similar to those of the conventional uniaxial creep test of the white sandstone. The instantaneous strain at all stress levels of the damaged rock samples increased gradually with the stress level, which is consistent with the non-linear functional relationship; Based on the time-dependent damage effect, the two-parameter Weibull distribution function was introduced into the West Plains viscoelasticity model, and a creep damage model for white sandstone was proposed. The improved Nishihara model can simulate the uniaxial creep characteristics of each damaged rock sample at various stress levels.

## Introduction

Energy resources (coal, metal ore, oil, gas, etc.) are an essential pillar of the rapid development of the world economy. The shallow parts of the earth are exhausted and resource exploitation is gradually moving to the deeper parts. Xie et al.^1^. Due to the unique characteristics of the environment and the complexity of the stress field, especially in the "three high and one disturbed" environment of the deep rock body, the strength of the surrounding rock before excavation is low, already in a latent plastic or plastic state, the mining of mineral resources is the unloading process of the rock body^2,3^. The mining pressure caused by the extraction of mineral resources will lead to damaged perimeter rock, further weaken its mechanical properties and the structure of the surrounding rock. However, this is limited by the underground works' characteristics, the damaged surroundings' mechanical properties, and the need to use support structures to form a standard bearing body to control large deformations and failing surrounds^4,5^. Therefore, an in-depth study of the rheological properties of damaged surrounding rocks is essential to evaluate their pre-peak mechanical properties correctly and explain the mechanisms by which they experience enormous rheological deformation damage, thus propose efficient and economical rock control techniques for deep engineering^6,7^.

Domestic and international experts and scholars have extensively investigated the mechanical properties of rocks under unloading. Tunnel excavation in a high ground stress environment causes strong stress redistribution, resulting in unloading damage to the surrounding rock^8–10^. As the damaged surrounding rock is the main load-bearing body during the tunnel operation, the full stress–strain curves of different medium rock types were obtained by rigid rock mechanics testing machines to reveal the mechanical properties of the unloading damaged surrounding rock body^11^. With the rise of fractography, Fu et al.^12^ studied the time-varying behavior of fracture modes on a mesoscale. Zhao et al.^13^ investigated the effect of impact loading on the crack extension characteristics of coal samples. Liu et al.^14^ and Chu et al.^15^. noted the microscopic mechanisms of brittle creep in saturated rocks, and studying of their mechanical behavior after creep damage is vital for rock engineering design and predicting the long-term evolution of the Earth's crust. Javadi et al.^16^ found that numerical analysis serves as a bridge between the constitutive model and the data, and its inverse analysis method also plays a crucial role in discerning the reliability of the constitutive model (thesis). Yang et al.^17^ investigated the mechanism of fine-scale damage in short-time creep. Lin et al.^18^ found that chemical corrosion significantly affects the damage evolution behavior of rocks. Cao et al.^19^ based on the nonlinear damage creep characteristics of rock and damage variable, new nonlinear damage creep constitutive model of high-stress soft rock is defined in series with the improved Burgers model, Hooke model, and St Venant model.

Extensive literature studies creep damage mechanisms in pre-peak unloading damage and time-damaged rocks. While these are important, only a few comprehensive studies have considered both factors. Moreover, there are limited reports on the long-term strength of rocks, which is an essential parameter for characterizing their mechanical properties. Therefore, in this study, we seek to experimentally and theoretically assess the time-dependent deformation behavior and damage properties of white sandstone with pre-peak unloading damage. By comparing the fracture morphology and crack extension in creep tests of pre-peak unloading damaged white sandstone to analyze the exemplary mechanism of creep rupture of white sandstone under different degrees of damage. According to the test results, a variable parameter creeps damage model is proposed, and the model accounts for the effects of the pre-peak unloading damage on white sandstone.

## Materials and methods

### Materials and sampling

**Figure 1:**
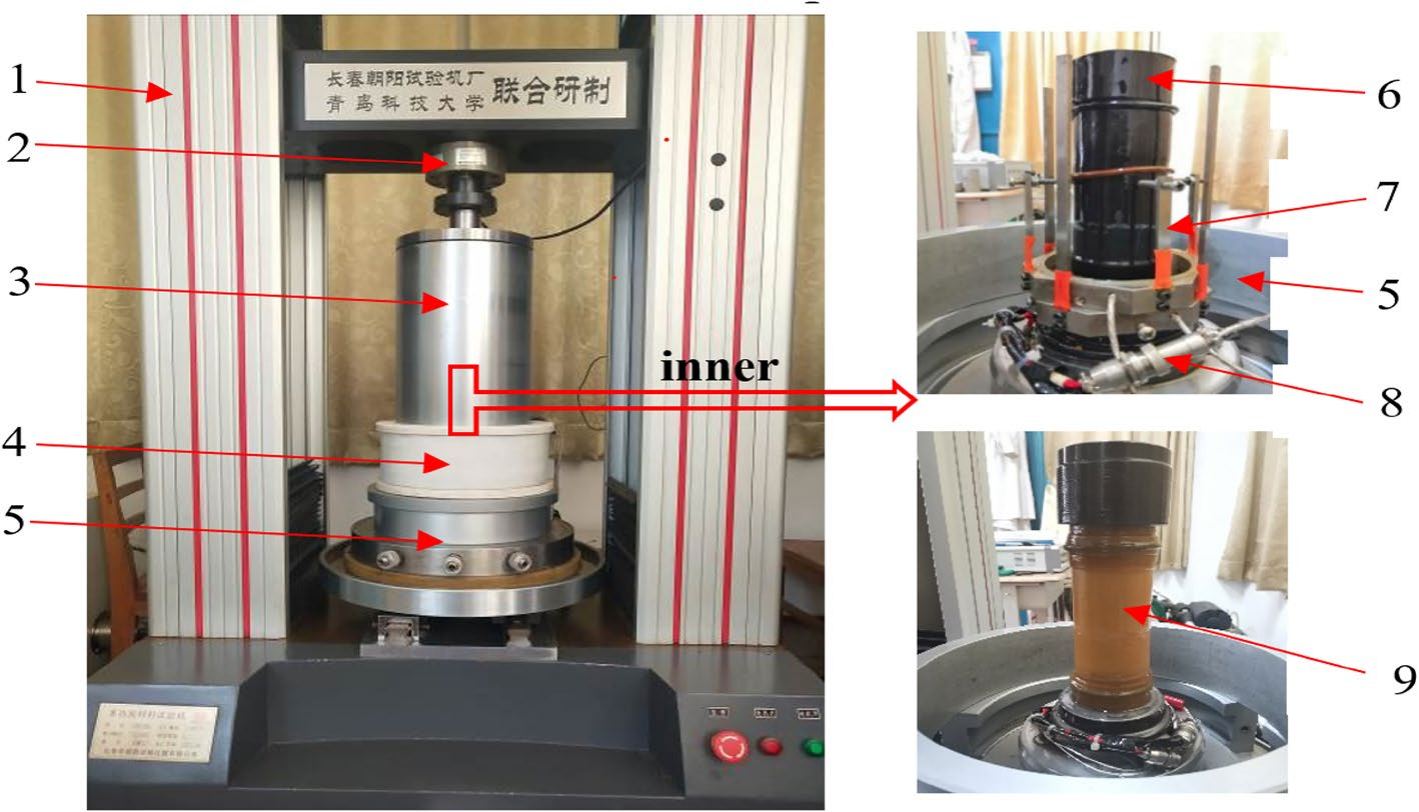
Triaxial compression test: 1—testing machine frame, 2—pressure sensor, 3—confining pressure chamber, 4—electromagnetic heating coil, 5—surrounding block, 6—heat shrinkable tube, 7—Radial displacement sensor, 8—temperature sensor, 9—rubber tube.

For the conventional uniaxial creep test and the peak front unloading damage rock sample preparation test, the TAW-200 electronic multifunctional material mechanics tester (see Fig. 1) was designed by Changchun Chaoyang Testing Machine Factory in conjunction with the Mechanics Teaching and Research Department of Qingdao University of Science and Technology, its main performance indicators are as follows: the maximum vertical static load of 200 kN, system accuracy < 0.5%; maximum compression deformation of 20 mm; Hooke pressure chamber bearing 30 MPa; servo-hydraulic stroke of 50 mm. Specimen size of *Ф*50mm × 100 mm, *Ф*75mm × 150 mm two specifications.

**Figure 2:**
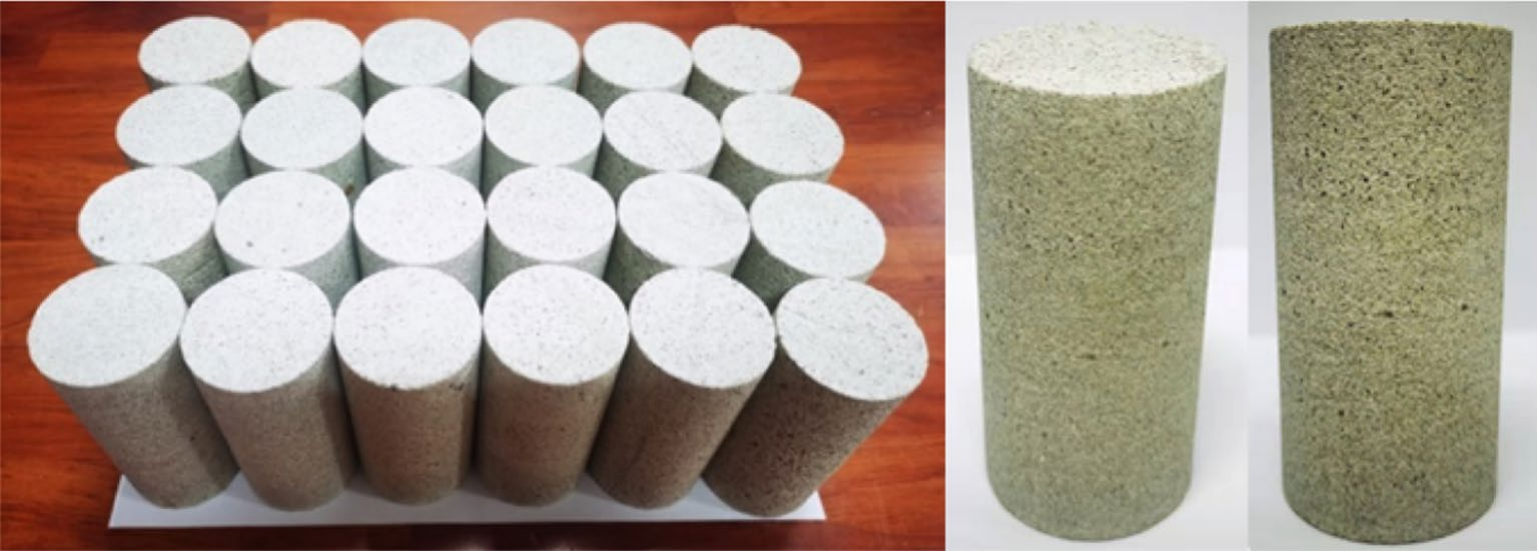
Rock white sandstone specimens for tests.

White sandstone specimens taken from the mountains of Zigong City, Sichuan Province, take out the core by the rock automatic coring machine and double-end grinding machine to prepare a standard rock sample of Ф50mm × 100 mm (see Fig. 2).

**Figure 3:**
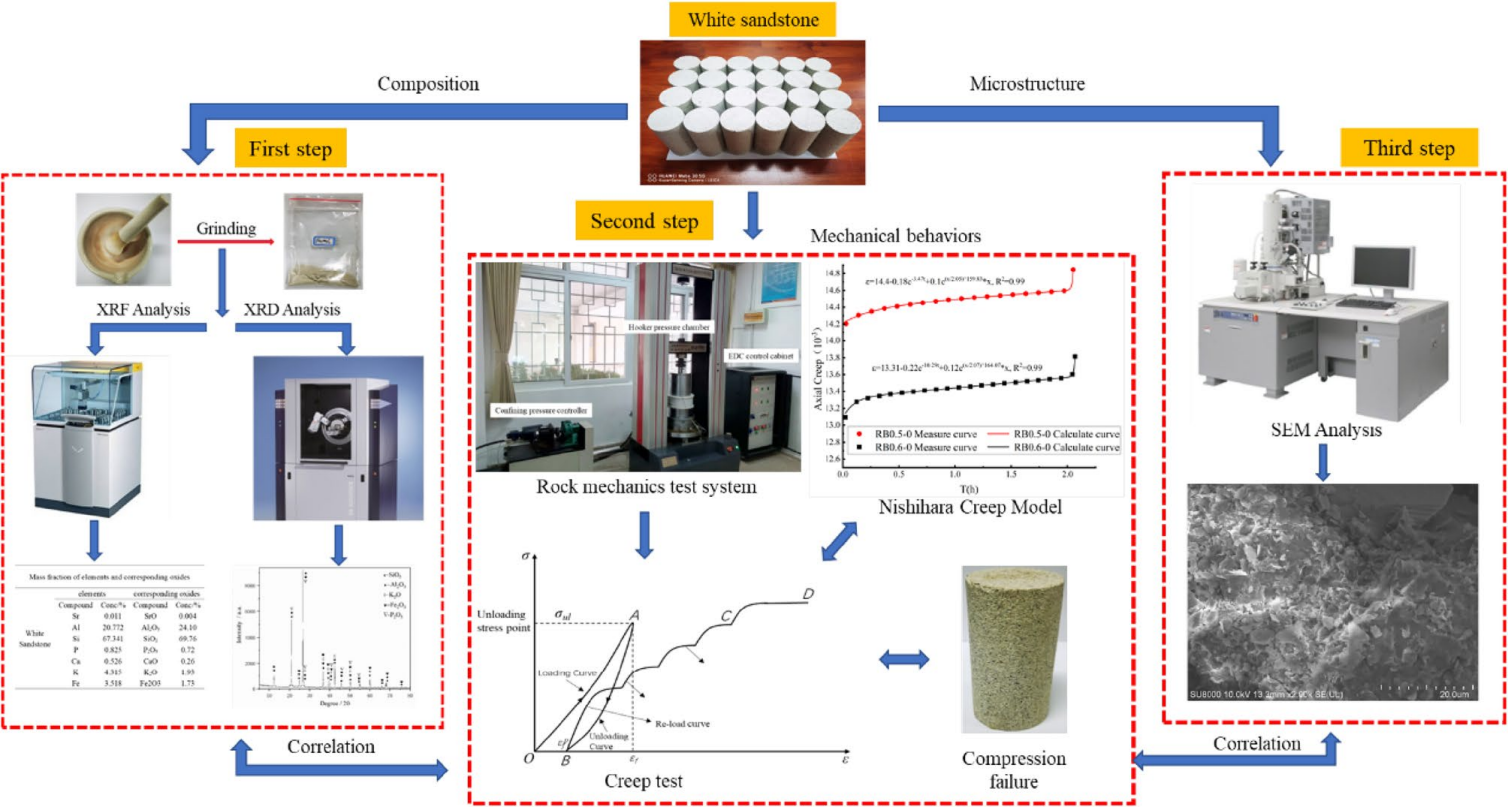
Experimental scheme for macro- and mesoscopic failure and uniaxial creep testing of white sandstone.

To reduce the influence of the inhomogeneity of rock-like materials on the test results, the NM-4A/B non-metallic ultrasonic flaw detector was used to perform nondestructive ultrasonic flaw detection on standard cylindrical specimens, and standard white sandstone specimens with close acoustic velocities were selected for subsequent tests. XRD and XRF tests were then used to determine the material properties of the white sandstone specimens, mainly composed of quartz, clay, and acerulite. Among them are ~ 69.76% of quartz α-SiO_2_, ~ 24.1% of clay $\text{Al}_{2}\text{O}_{3}\cdot 2\text{SiO}_{2}\cdot {2\text{H}}_{2}\text{O}$, ~ 1.73% of acanthite Fe_2_O_3_, and ~ 0.004% of calcite S_r_O. The effect of creep damage on the macroscopic creep mechanical properties after unloading damage was evaluated by scanning electron microscopy (SEM) of creep-damaged fractures of white sandstone, providing a theoretical basis for the long-term stability evaluation of the construction demonstration. The final rock sample details are shown in Table 1, and the test procedure is shown in Fig. 3.

**Table 1:** Details of prepared rock samples.

Number	Unloading point/%	Specimen size		Quality/g	wave speed /m s^-1^	Test plan
		Diameter/mm	Height/mm			
DR0-0	0	49.1	103.16	432.85	2212	Uniaxial Creep Test
DR1-0	50	51.6	99.46	441.73	2170	Tri-axis peak front loading and unloading + Uniaxial Creep Test
DR2-0	60	49.92	102.97	462.26	2140	
DR3-0	70	50.4	102.06	442.3	2160	
DR4-0	80	50.22	102.38	455.87	2000	
DR5-0	90	49.44	101.35	463.32	2050

### Experimental process

**Figure 4:**
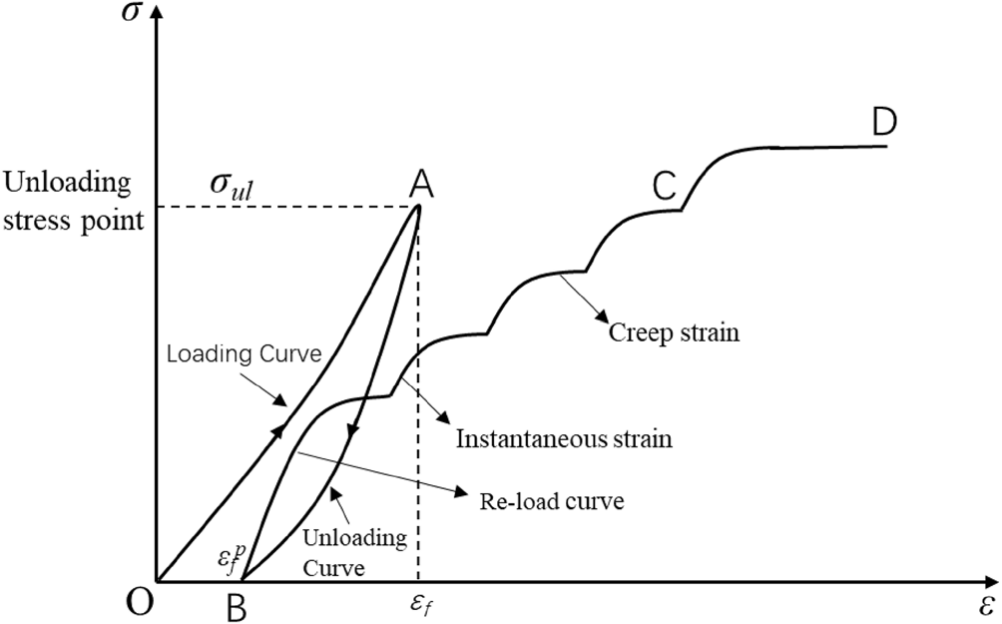
Schematic diagram of pre-peak unloading damaged rock sample preparation and the reloading test.

Under the strong unloading disturbance of deep high stress roadway excavation, it will inevitably lead to damage and deterioration of the surrounding rock. After roadway excavation, the stress of the surrounding rock will be redistributed, the stress state will change, and the surrounding rock will continue to be damaged until destroyed, which will pose a serious threat to the stability of the surrounding rock. Therefore, based on the triaxial loading unloading test, damaged rock samples are obtained (see Fig. 4). Then the uniaxial creep test of the pre-peak unloading damage white sandstone was carried out.

The first stage is a triaxial compression test of an intact rock sample (see section OAB in Fig. 4). Martin points out that after the rock load is loaded to 40% of the peak strength^20,21^, and damage is generated. In Fig. 4, ${\sigma }_{\mu l}$ is the unloading stress point before the peak, ${\varepsilon }_{f}$ is the unloading strain, ${\varepsilon }_{f}^{p}$ is the residual strain. The specific steps are as follows: Firstly, carry out the triaxial compression experiment of the complete specimen at ${\sigma }_{3}$ = 1 MPa, unloading at the peak stress points ${\sigma }_{\mu l}$ = 0, 50%, 60%, 70%, 80% and 90% before the peak.

The second stage is the creep test of peak front unloading damaged white sandstone (see section BCD in Fig. 4). The creep in this test was set with multi-stage loading levels, the initial load value was 50% of the uniaxial compressive strength value, and the subsequent graded load increments were 5 kN (2.5 MPa). The test method was the same as the conventional uniaxial compression test, the loading method was displacement loading, and the loading rate was 0.2 mm/min. The objective is to obtain time-strain curves for the pre-peak unloading damaged white sandstone and to provide basic data for analyzing the creep mechanical properties of the pre-peak unloading damaged rock samples.

The prepeak unloading damage variable is calculated by the elastic modulus method^10^, which can obtain the damage variable of the prepeak unloading damage test more accurately by coupling plastic deformation and the damage mechanism:

1 $$ D = 1 - \left( {1 - \frac{{\varepsilon^{r} }}{\varepsilon }} \right)\frac{{E^{r} }}{{E_{0} }} $$

**Figure 5:**
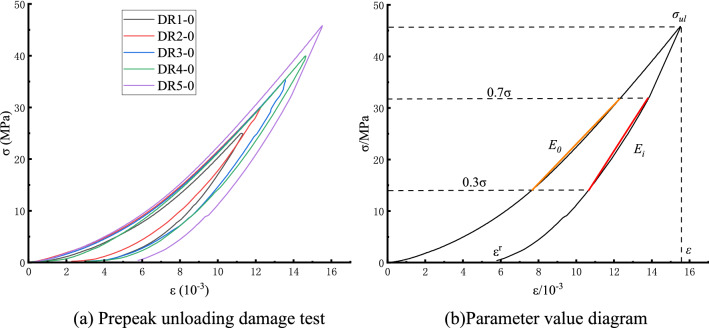
Stress–strain curve of the prepeak unloading damage.

**Figure 6:**
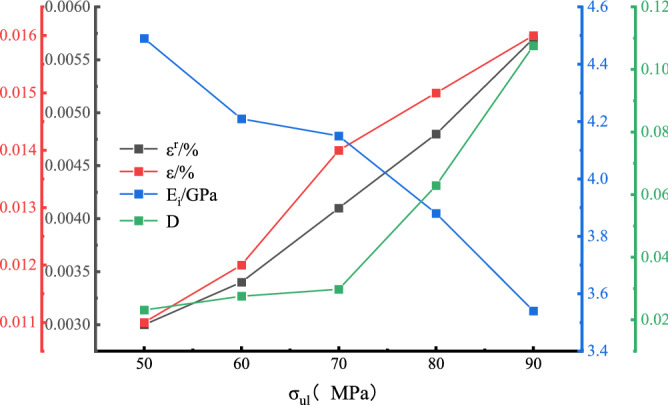
Variation curve of reload deformation characteristics with unloading point.

In this formula, $D$ is the variable of the peak unloaded injuries, $\varepsilon^{r}$ is the residual strain, $\varepsilon$ is the total strain, $E_{i}$ is the unloading flexible modulus, and $E_{0}$ is the initial elastic modulus. The modulus of the elastic modulus ranges from 30 to 70%^10^ in this paper. The selection of each parameter is shown in Fig. 5, and the calculated injury variable and residual strain conversion curve are shown in Fig. 6, where the unloading point $\sigma_{ul}$ is the unloading strength and the peak intensity ratio.

Figure 6 shows that the damage variables and residual strain are positively correlated with the change in the unloading point; with the increase in the unloading point, the damage variable increases to 0.0232, 0.0276, 0.0297, 0.0629, and 0.1076, and the residual strain increases to 0.003, 0.0034, 0.0041, 0.0048, 0.0057 respectively.

## Results and analysis

### Creep test results

The deformation of rocks not only exhibits elasticity and plasticity, but also has rheological properties, which include creep, relaxation and elastic after-effects^22,23^. Six sets of uniaxial creep tests were conducted for this peak front unloading damaged white sandstone, with each level of horizontal stress maintained for 4 h during the trial. A single test's maximum creep test time can be up to 28 h. Before the creep test, it is known from the conventional test data of the white sandstone that the uniaxial compressive strength is ${\sigma }_{c}$ = 40 MPa, elastic modulus *E* = 4.65 GPa, Poisson's ratio *ν* = 0.21, cohesion *c* = 9.82 MPa, and internal friction $\varphi$ = 55.63°.

**Figure 7:**
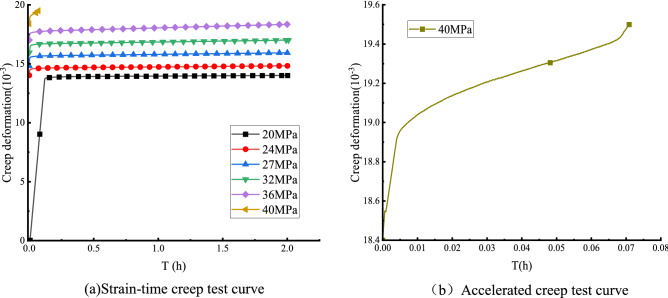
Uniaxial creep curve of non-destructive white sandstone specimen.

**Figure 8:**
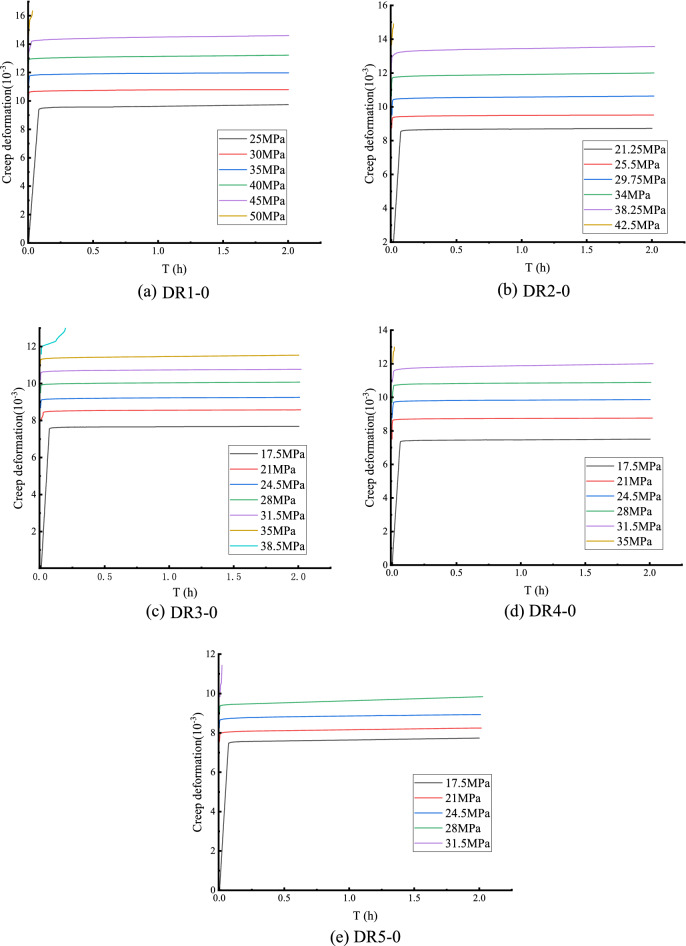
Creep curve of white sandstone with pre-peak unloading damage.

Boltzmann superposition method was used to process the creep data of pre-peak unloading damaged white sandstone in stages to obtain creep curves of white sandstone with different degrees of pre-peak unloading damage, and the nondestructive white sandstone creep curves are shown in Fig. 7. The uniaxial creep test curves of peak front unloading damaged white sandstone are demonstrated in Fig. 8.

Figure 7 shows that the creep deformation of white sandstone has good continuity. When the stress level is elevated, there will be an apparent transient stress deformation phenomenon, the curve change is a nearly straight line, after that there will be creep phenomenon of white sandstone specimens with the continuous action of stress. Figure 7a,b show that: when the non-destructive specimen is subjected to stress level $\sigma$ = 40 MPa, the uniaxial creep curve of the rock at all levels of stress level only appears at the deceleration creep stage, equal creep stage, i.e., the rock undergoes stable creep; when the non-destructive specimen reaches the creep stress threshold $\sigma$ = 40 MPa, the specimen curve appears the typical creep three stages: deceleration creep stage, isometric creep stage, accelerated creep stage, that is, the specimen undergoes unstable creep. Stable creep or unstable creep depends on the current stress state, and when the specimen is subjected to high stress, complete creep will occur in three stages, the specimen will be destroyed after unstable creep occurs. When the creep stress value $\sigma$ = 40 MPa, creep damage strain value ε = 19.4378 × 10^–3^. It can be seen that the creep phenomenon is evident during the test.

**Figure 9:**
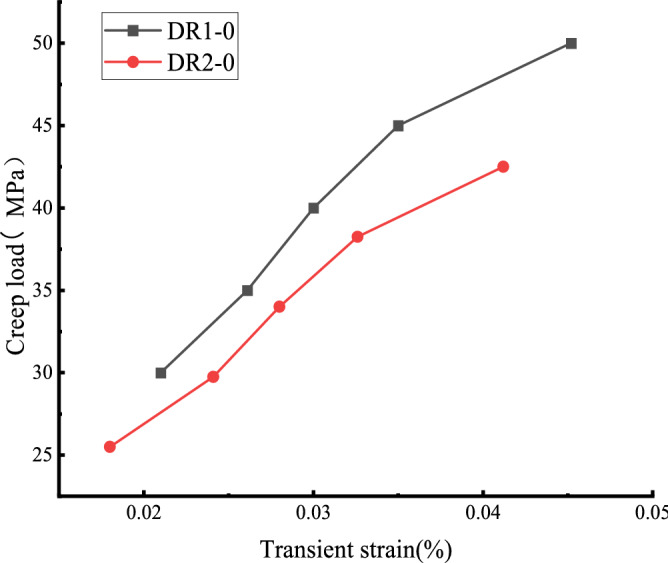
Relationship between instantaneous axial strain and creep loading.

Figure 8 shows that the creep curve of the white sandstone specimen with unloading damage before the peak increases in a step-like manner as a whole. The total creep strain consists of the instantaneous strain after the applied load and the creep strain under the constant load. The rapid deformation is mainly affected by the size of the creep load, the loading rate, and the rock properties. The axial transient strain's value increases with the degree of unloading damage before the peak. Figure 9 shows that the first-order instantaneous strain value of the DR1-0 test curve is 0.021%,and the transient strain value increases to 0.0261–0.0452% from the second to the fifth stage. The DR2-0 test curve shows that the instantaneous strain value of the first stage is 0.018%, and the instantaneous strain value of the second stage to the fifth stage increases to 0.0241–0.0412%. The creep stress threshold during the creep test affects the transient deformation, taking the DR1-0 curve as an example, it can be seen that when the creep load σ = 40 MPa, the transient strain in the creep curve of the white sandstone specimen changes from a nearly linear change to a nonlinear trend. At this time, there is an obvious inflection point in the creep curve of the specimen, which is due to the accumulated damage to the internal structure caused by the specimen undergoing pre-creep action with continuous.

**Figure 10:**
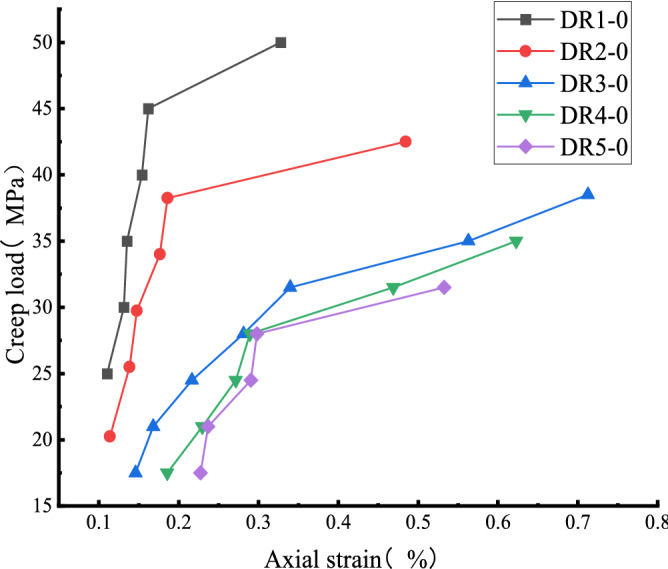
Relationship between creep strain and creep load of sandstone.

Figure 10 and Table 2 show that the uniaxial creep strain of white sandstone gradually increased from 0.1104 to 0.32764 with the increase in stress level. The creep strain increased significantly when the stress level exceeded the stress threshold. The creep strain increased by 100% at the sixth level compared to the fifth. Compared with the DR2-0 and DR3-0 test curves, the uniaxial creep strain increases from 0.1137 to 0.186 and 0.146 to 0.3396 respectively. Overall, the rock's axial creep at all stress levels is in line with the nonlinear trend. The pre-peak unloading damage effect leads to the development of primary micro-defects in the internal structure of the specimen. The specimen is in a non-stable state compared with the intact specimen. When the stress level of the specimen is lower than the stress threshold, the non-steady-state changes to the steady-state, which is manifested by the occurrence of steady creep with a small amount of axial creep; when the stress level is greater than the stress threshold, the internal structure of the specimen undergoes structural transformation, which is manifested by the occurrence of unsteady creep, and finally until the rock structure is destroyed.

**Table 2:** Creep strain of sandstone under different stress levels.

Serial number	Creep strain (10^–3^)						
	Level 1	Level 2	Level 3	Level 4	Level 5	Level 6	Level 7
DR1-0	0.1104	0.1312	0.1352	0.1539	0.1618	0.32764	
DR2-0	0.1137	0.1384	0.1474	0.1764	0.186	0.4842	
DR3-0	0.146	0.1682	0.2166	0.2812	0.3396	0.5628	0.7127
DR4-0	0.1856	0.2295	0.2715	0.2892	0.4687	0.6626	
DR5-0	0.2273	0.2367	0.2904	0.298	0.5326		

**Figure 11:**
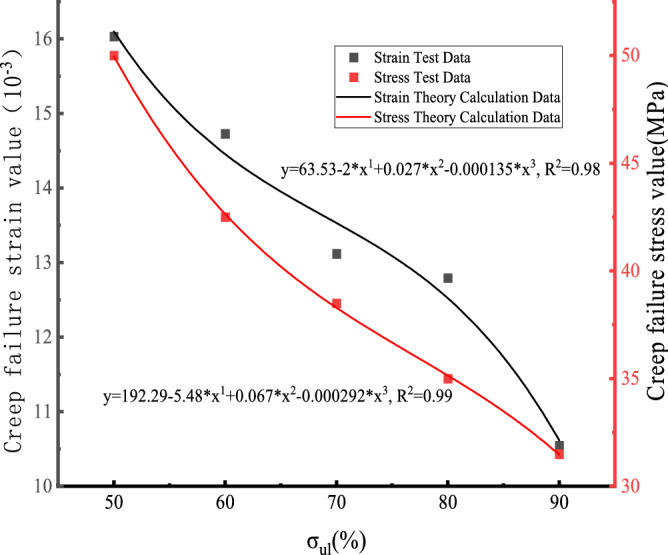
Creep failure stress–strain curve of white sandstone.

The uniaxial creep test data of white sandstone with pre-peak unloading damage are compiled in Fig. 11. Figure 11 shows that the creep damage stress–strain values all decrease with the increase in the degree of pre-peak unloading damage of the rock, and they both show a significant nonlinear relationship. The fitted creep damage strength curves for white sandstone are:

2 $$ \sigma = 192.29 - 5.48 \cdot \sigma_{ul} + 0.067 \cdot \sigma_{ul}{^{2}} - 0.000292 \cdot \sigma_{ul}{^{3}} $$

From the fitted equation given in the figure, it can be calculated that when the degree of pre-peak unloading damage is $\sigma_{\mu l}$ = 50%, the theoretically calculated creep damage stress is 49.29 MPa, which is about 98% of the sandstone test data. However, when the pre-peak unloading damage degree $\sigma_{\mu l}$ = 90%, the creep damage stress is much less than 31.5 MPa, about 28.922 MPa, which is approximately equal to the experimental data.

**Figure 12:**
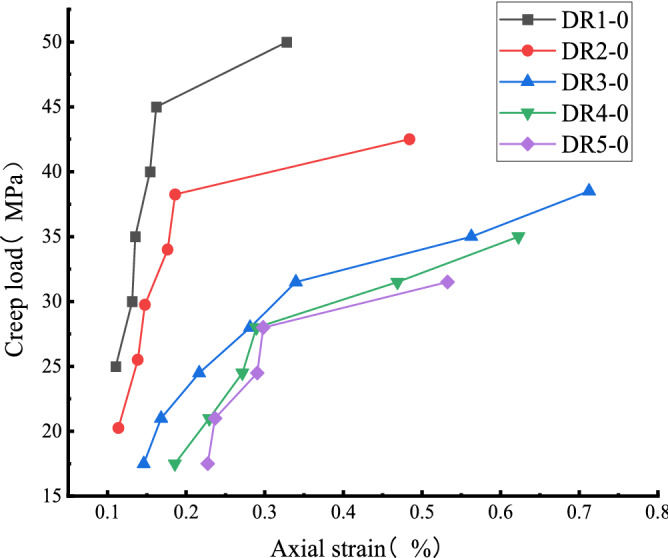
Relationship between steady state creep rate and creep stress.

The creep rate of a rock is a physical quantity that characterizes how fast it deforms under a constant load, and it is also an important index for studying the creep properties of rocks^24^. Figure 12 shows that the steady-state creep rate increases nonlinearly with the increase of creep load. Taking the DR1-0 curve as an example, when the stress level is small, that is, the stress σ < 45 MPa,the steady-state creep rate of the peak front unloading damaged white sandstone specimen changes less, and the steady-state creep rate gradually increases from 0.058 to 0.085 (10^–4^/h), but when the specimen is in a high-stress state, that is, $\sigma \ge$ 45 MPa, the steady-state creep rate changes from a linear to a non-linear trend, and the steady-state creep rate is 0.17 (10^–4^/h), which is 100% higher than the steady-state creep rate of the previous level; finally, the specimen is damaged by creep.

### Long-term strength analysis

**Figure 13:**
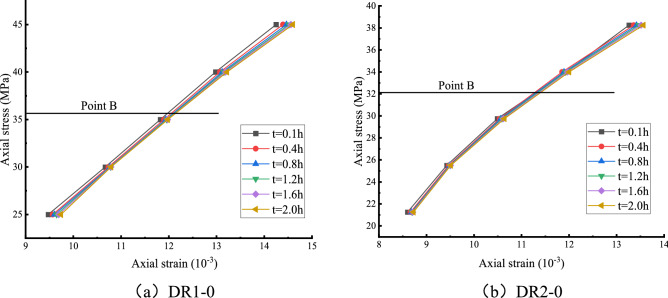
Isochronous stress–strain curve.

The isochronous curve method involves transforming the straight line of each isochronous line into a curve, similar to the stress value corresponding to the asymptote formed by the yield stress is the long-term strength of the rock^25^. The inflection point of each isochronous line marks the transformation of the rock from the viscoelastic stage to the viscoplastic stage, and the internal structure of the rock changes and begins to break down. Figure 13 shows the isochronous stress–strain diagrams for the two curves DR1-0 and DR2-0.

It can be seen in Fig. 13 that there is point B in the stress–strain isochronous curve cluster. Point B is the critical point of the transformation from viscoelastic deformation to viscoplastic deformation of the rock sample, and the corresponding stress value is the long-term strength of the rock sample.

Table 3 shows that: (1) The creep damage strength and long-term strength of the rock decreased as the degree of unloading damage before the peak increased, while the long-term creep strength of the white sandstone specimen was lower than its creep damage strength. (2) According to the test data, under the action of constant stress for a long time, the rock would be damaged at a lower strength than the creep damage strength, and the long-term strength/damage strength ratio was about 0.7–0.8.

**Table 3:** Long-term strength of white sandstone damaged by unloading before peak.

Serial number	Instantaneous strength/MPa	Long-term strength/MPa	Long-term strength/instantaneous strength
DR1-0	50	35.607	0.712
DR2-0	42.5	32.083	0.755
DR3-0	38.5	27.019	0.701
DR4-0	35	25.655	0.733
DR5-0	31.5	22.554	0.716

**Figure 14:**
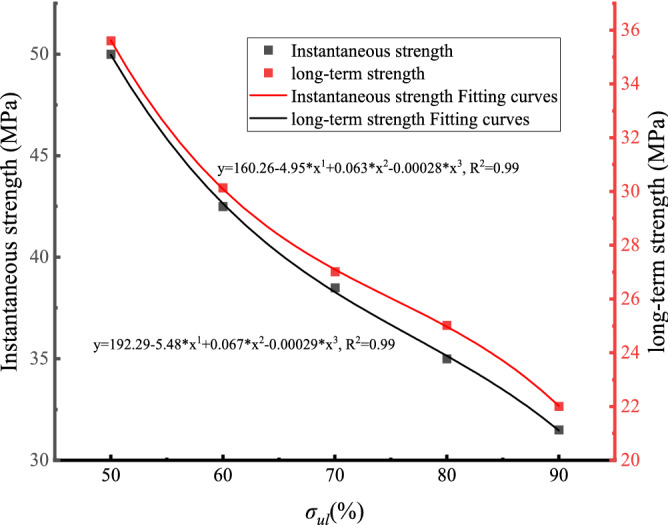
Long-term strength and failure strength fitting curve.

Figure 14 shows that the overall reduction in long-term strength of the rock samples was 36.7%. This indicates that the effect of pre-peak unloading damage has a greater impact on the long-term strength of the rock samples. The peak front unloading damage and creep stress damage are mutually reinforcing, and the coupling of the two effects is much more damaging to the rock than the effect of the two factors alone. As can be seen from the fit, the variation of both is consistent with the non-linear empirical equation^26^. The long-term strength fitting equation:

3 $$ \sigma_{L} = 160.26 - 4.95 \cdot \sigma_{{{\text{ul}}}} + 0.063 \cdot \sigma_{{{\text{ul}}}}{^{2}} - 0.00028 \cdot \sigma_{{{\text{ul}}}}{^{3}} $$

## Discussion

### Failure and deterioration mechanism of white sandstone

**Figure 15:**
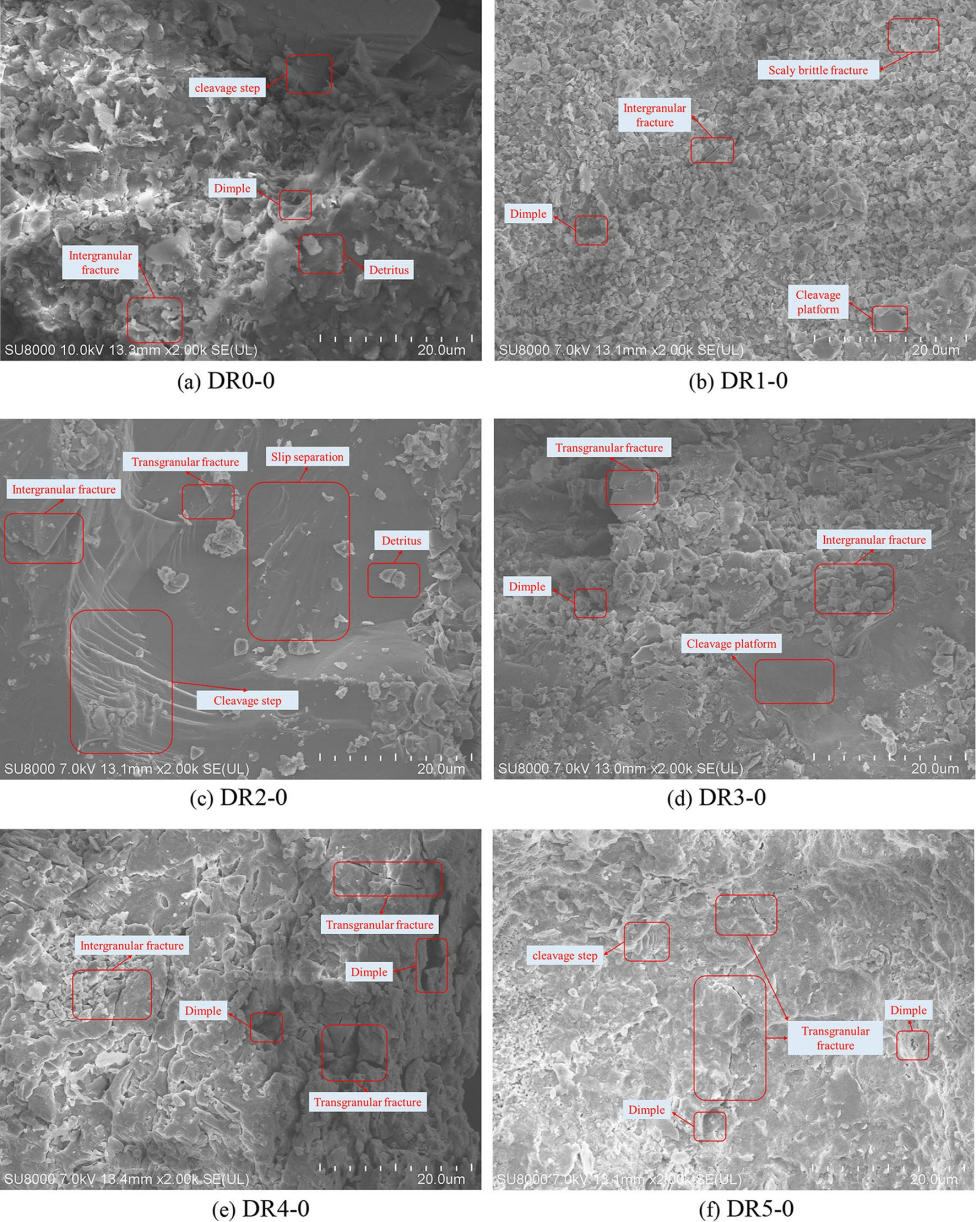
Mesoscopic failure structure of white sandstone (×2000).

Rock is a complex mineral collection with its own particular internal material composition and spatial structure^27^. The fracture extension direction, fine fracture characteristics, and the relative displacement direction of rock masses on both sides of the section were judged by the morphological characteristics of different fractures^28^. By comparing the fracture morphology and fracture extension of creep specimens of peak-unloading damaged white sandstone, the creep damage mechanism of peak-unloading damaged white sandstone is analyzed. Microscopic analysis adopts the Hitachi High-tech SU8000 Scanning Electronic Microscope, and the SEM images of white sandstone fracture with varying damage is shown in Fig. 15.

Figure 15a–f show the scanning electron micrographs of creep fracture at 2000 magnification for the nondestructive specimen and the pre-peak unloading damaged specimen of white sandstone respectively. The fine fracture observation surface of the nondestructive specimen shows obvious deconstruction steps, and the fracture joints of each deconstruction platform are relatively flat. In addition, a small amount of crystal debris is present on the fracture observation surface. Figure 15b shows that the fracture observation surface is rough, obvious scale-like brittle fractures and along-crystal cracks can be observed, and a few tough nests are produced on the fracture surface. Figure 15c shows that the fine view fracture observation surface of the pre-peak unloading damaged white sandstone also shows an obvious deconstruction platform. The fracture observation surface is relatively flat with only a few crystal debris particles.

In contrast, the deconstruction platform's extension direction is consistent with the deconstruction damage on the fracture observation surface. Figure 15d shows that the brittle-tough transition phenomenon begins to appear in the fine fracture morphology. The fine fracture observation surface has obvious tough nest groups and is rougher. Figure 15e shows that the fracture surface has obvious transgranular cracks in the low concave area. The along-crystal cracks and transgranular cracks coexist and develop together. Figure 15f shows that many tough nests are distributed on the fine fracture observation surface. The transgranular cracks and along-crystal cracks intersect, forming an obvious macroscopic fracture zone on the fine fracture observation surface of the specimen.

In the creep process of white sandstone, the internal crystal structure of the rock is in the joint action of constant axial force and peak front unloading damage effect; when the damage degree is low, the relative displacement degree between crystals is low. During the relative motion of the crystal particles, the incompatibility of the deformation of the crystal particles will cause local stress concentration and then form obvious deconstruction platforms. When the degree of damage is higher, the dislocations in the internal structure of the crystal increase rapidly, the crystal particles' strength decreases, and cracks form transgranular cracks from inside the crystal. Under the continuous effect of long-term stress, the crystal particles are not in equilibrium due to the pre-peak unloading damage effect, and the cracks develop rapidly. The fracture observation surface shows that the transgranular cracks and along-crystal cracks are staggered through. Overall, with the increase of the pre-peak unloading damage degree, the internal cracks of the white sandstone specimens develop chaotically, the meso-structure damage intensifies, and the overall performance is more "soft" due to the weakening of local properties. It can be seen that the white sandstone experiences a higher pre-peak unloading damage effect, the damage caused by creep is more complex, and the cracks are more abundant.

The deformation and strain of the white sandstone with pre-peak unloading damage were measured by PMLAB DIC-2D, and the moment of failure of the specimen was selected after shooting, the transient failure form of the specimen is shown in the upper part of Table 4. Using the crack detection system for rock specimens based on Matlab-GUI, several real-time damage crack detection tests for white sandstone specimens were carried out. The image processing results are shown in the lower part of Table 4.

**Table 4:**
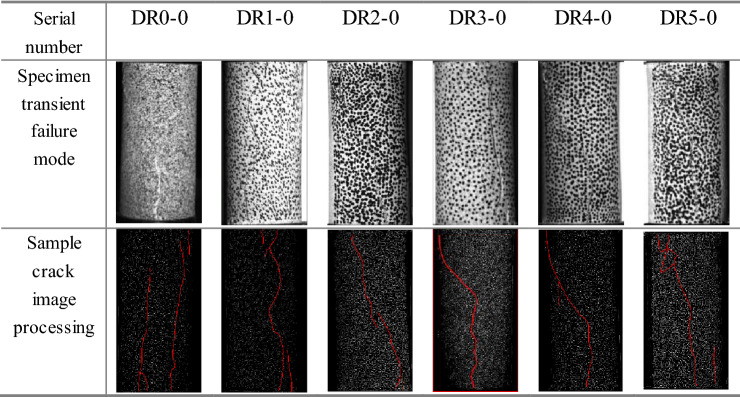
Macroscopic damage characteristics of pre-peak unloading damaged white sandstone.

Table 4 shows that the white sandstone in the nondestructive natural state exhibits splitting tensile damage, and the damage form of white sandstone gradually changes from "splitting"-"single bevel shear damage"-"splitting-shear damage" with the increase of unloading damage. DR0-0 images show that multiple cracks are generated at the rupture initiation point of the specimen, and the expansion of the primary cracks triggers the generation of secondary cracks. DR1-0 images show that the damage of the specimen is in the form of splitting damage, which leads to the destruction of the rock end by dilation phenomenon. DR2-0 and DR3-0 images show that the damage of the specimen is in the form of single bevel shear damage, a main crack penetrates the whole specimen from top to bottom, and the fracture angles of the specimen are about 68° and 71° respectively, and the overall shape of the specimen remains basically intact. DR4-0 image shows that the specimen still exhibits significant shear damage with a primary shear fracture crack angle of 72° and secondary cracks appears in the middle of the rock specimen. DR5-0 image shows that the specimen damage is in the form of mixed shear-splitting damage with a primary crack angle of 70°.In addition, splitting cracks are generated from the top and eventually intersect with shear cracks, and there are fewer secondary cracks at the top of the specimen.

**Figure 16:**
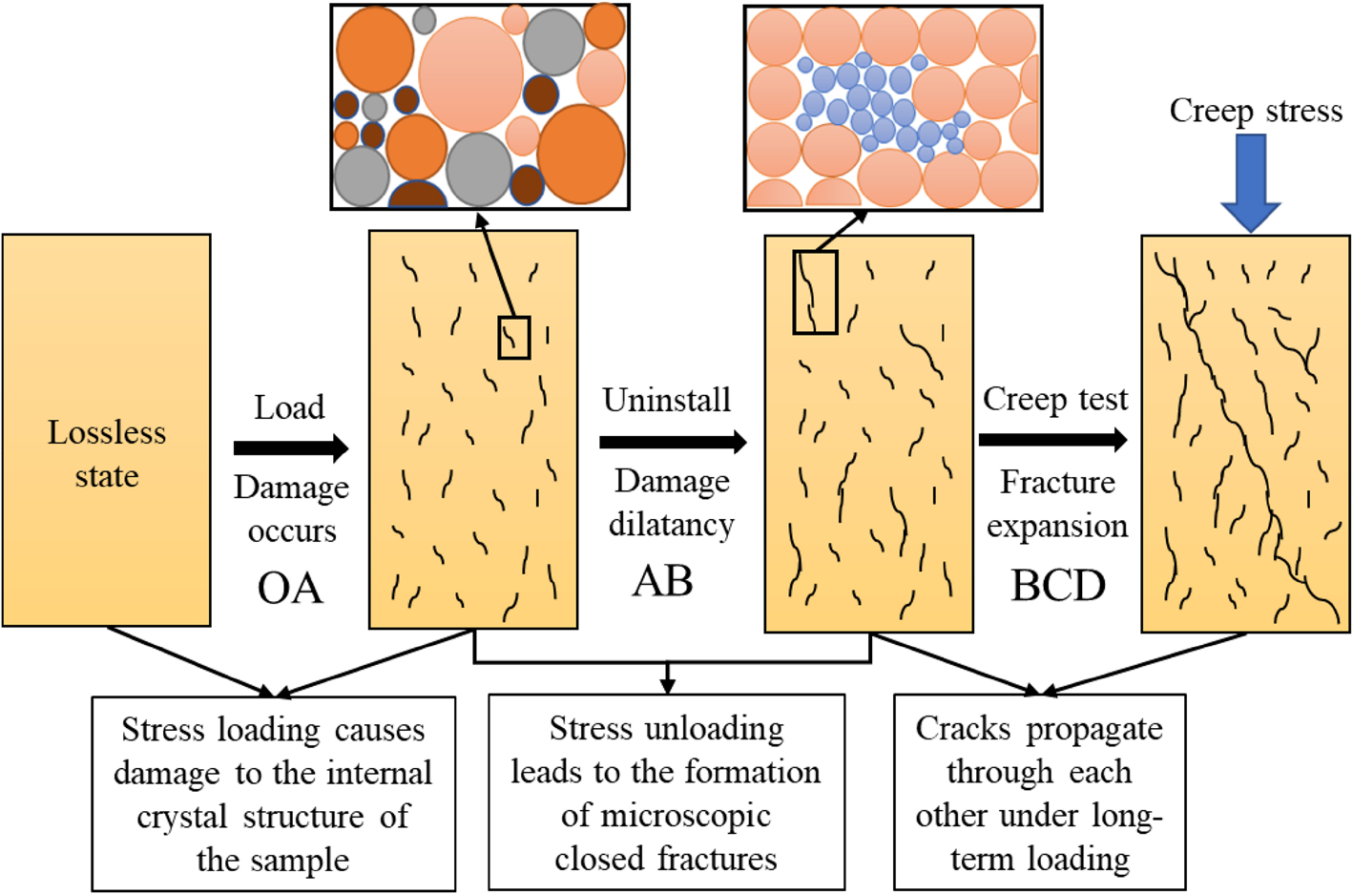
Damage and fracture evolution processes.

The macroscopic damage morphology of white sandstone damaged by pre-peak unloading is significantly different, and the reason is attributed to (1) The mineral composition of the white sandstone contains a large number of clay minerals, such as montmorillonite, kaolinite and chlorite, etc. Different minerals have uneven deformation under the action of pre-peak unloading damage, causing certain damage to the sample; (2) The microscopic closed cracks in the white sandstone gradually accumulate inside the sample, and under long-term loading conditions, aging damage helps the new cracks and the original cracks to penetrate each other to form a macroscopic fracture zone. The propagation of the cracks in the lower specimen will gradually stabilize. Under the condition of yield stress, due to the reduction of the effective area, a stress concentration effect is formed inside the sample, the stress exceeds the strength of the rock micro-element, and the micro-cracks continue to develop and extend, and finally the sample ruptures along the main failure surface (see Fig. 16).

The above test results show that the pre-peak unloading damage white sandstone creep test shows obvious weakening phenomenon, and the pre-peak unloading damage effect involves all stages of rock creep. In order to further study the damage effect of white sandstone in each creep stage, from the perspective of rock creep constitutive relationship, a creep constitutive model considering the pre-peak unloading damage effect is constructed to further quantitatively explore the pre-peak unloading damage. Creep properties of sandstone under action.

### Creation and analysis of damage components

The effect of pre-peak unloading damage on rock creep characteristics can be qualitatively expressed as under the constant stress of external load, the rock attenuation creep, isokinetic creep and accelerated creep all change with the weakening effect of pre-peak unloading damage^29^.

The quantitative expression can be based on the non-quantitative characteristics of rock creep mechanical parameters changing with the weakening degree of pre-peak unloading damage, and the long-term creep action of rock will cause fatigue damage, and the parameters based on aging characteristics themselves have attenuation. Therefore, in terms of macroscopic mechanical behavior, this parameter can describe the coupling effect of pre-peak unloading damage and creep damage.

First, considering that the viscoelastic parameters have unsteady eigenvalues that vary with pre-peak unloading damage, the details are shown below.

4 $$ E(U) = K_{e} (U)E_{0} $$

5 $$ \eta (U) = K_{\eta } (U)\eta_{0} $$

where: $E(U)$, $\eta (U,t)$ is the elastic model and viscosity coefficient after the weakening of the pre-peak unloading damage; $E_{0}$, $\eta_{0}$ is the elastic modulus and viscosity coefficient of the rock in the nondamaged state; $K_{e}$, $K_{\eta }$ is the elastic modulus damage coefficient and the viscosity damage coefficient of the pre-peak unloading damage effect.

During long-term creep, the difference in long-term mechanical properties between microscopic crystal particles reflects that the accumulation of microscopic damage has a random distribution characteristic based on the aging effect, which is expressed by a statistical continuous distribution function as:

6 $$ dD/dt = f(t) $$

where, $f(t)$ is the damage density function; $D$ is the long-term load damage variable based on aging effect; Considering that the aging damage accumulation is a nonlinear incremental behavior, a two-parameter Weibull distribution function is introduced to define the damage density function i.e.:

7 $$ f(t) = \frac{\theta }{\lambda }\left( {\frac{t}{\lambda }} \right)^{\theta - 1} e^{{ - (t/\lambda )^{\theta } }} $$

8 $$ D_{R} = \int_{0}^{t} {\frac{\theta }{\lambda }\left( {\frac{t}{\lambda }} \right)^{\theta - 1} e^{{ - (\frac{t}{\lambda })\theta }} } dt = 1 - e^{{ - (t/\lambda )^{\theta } }} $$

where, $\theta$, $\lambda$ are long-term load damage parameters.

The viscosity coefficient is an aging parameter with pre-peak unloading damage effect, so the variation law of viscosity coefficient has obvious aging attenuation and pre-peak unloading damage effect characteristics. load-damage coupling, and then the coupling equation can be obtained:

9 $$ \eta (U,t) = (1 - D)K_{\eta } (U)\eta_{0} = K_{\eta } (U)\eta_{0} e^{{ - (\frac{t}{\lambda })\theta }} $$

The above creep mechanical parameters are introduced into the Nishihara model, and the Nishihara damage constitutive model considering the damage effect of pre-peak unloading is established, which can better describe the uniaxial creep change law of white sandstone with pre-peak unloading damage.

### Creep modelling

**Figure 17:**
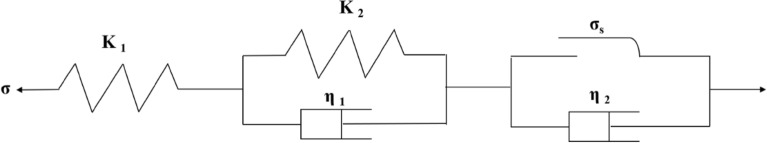
Nishihara Model Mechanical Model.

The Nishihara model consists of a Hooker body, a Kelvin body, and an ideal viscous body in series, which can fully respond to the elastic, viscous, and plastic properties of the rock^30^. Its mechanical model is shown in Fig. 17.

When $\sigma < \sigma_{s}$, the Y body is rigid, at this time the model rheological properties have creep properties. When $\sigma \ge \sigma_{s}$, at this time, the deformation of the creep model dissipates continuously with the increase of time. According to the series–parallel relationship between components in the previous section, it is known that $E(U)$ and $\eta (U,t)$ are introduced into the H-body and N-body in the West original model, while the damage due to the short-term effect is not obvious, so $\eta (U,t)$ is only introduced into the ideal viscoplastic body describing the accelerated creep stage. Based on the above content, a creep constitutive model of white sandstone considering pre-peak unloading damage was established:

10 $$\left\{\begin{array}{l}{\sigma }_{1}={\sigma }_{2}={\sigma }_{3}\\ \varepsilon ={\varepsilon }_{1}+{\varepsilon }_{2}+{\varepsilon }_{3}\\ {\sigma }_{1}={E}_{H}(U){\varepsilon }_{1}\\ {\sigma }_{2}={E}_{Y}\left(U\right){\varepsilon }_{2}+{\eta }_{Y}(U)\frac{d{\varepsilon }_{2}}{dt}\\ {\sigma }_{3}={P}_{t}\left(t\right)[{\sigma }_{s}+{\eta }_{N}(U,t)\frac{d{\varepsilon }_{2}}{dt}\end{array}\right.$$

where ${{\sigma_{1} } \mathord{\left/ {\vphantom {{\sigma_{1} } {\varepsilon_{1} }}} \right. \kern-\nulldelimiterspace} {\varepsilon_{1} }}$, ${{\sigma_{2} } \mathord{\left/ {\vphantom {{\sigma_{2} } {\varepsilon_{2} }}} \right. \kern-\nulldelimiterspace} {\varepsilon_{2} }}$ and ${{\sigma_{3} } \mathord{\left/ {\vphantom {{\sigma_{3} } {\varepsilon_{3} }}} \right. \kern-\nulldelimiterspace} {\varepsilon_{3} }}$ are the stress/strain corresponding to the Hooke body, Kelvin body, and ideal viscoplastic body in the West Plains model, respectively, $E_{H} (U)$ is the modulus of elasticity of the spring component in the Hooke body, $E_{Y} (U)$ is the modulus of elasticity of the spring component in the Kelvin body, $\eta_{Y} (U)$ is the coefficient of viscosity of the slider component in the Kelvin body, $\sigma_{s}$ is the creep yield strength, $\eta_{N} (U)$ is the coefficient of viscosity of the slider component in the ideal viscoplastic body, and $P(t)$ is the determination function, as follows:

11 $$ P(t) = \left\{ \begin{gathered} 0,t \le t_{s} \hfill \\ 1,t > t_{s} \hfill \\ \end{gathered} \right. $$

where, $t_{s}$ is the accelerated creep onset time point in the rock creep process. The first-order linear partial differential equation of formula (9) is obtained as

12 $$ \varepsilon (t) = \left\{ \begin{aligned} & \frac{\sigma }{{E_{H} (U)}} + \left( {1 - e^{{ - \frac{{\varepsilon_{Y} (U)}}{{\eta_{Y} (U)}}t}} } \right)\frac{\sigma }{{E_{Y} (U)}},t \le t_{s} \hfill \\ & \frac{\sigma }{{E_{H} (U)}} + \left( {1 - e^{{ - \frac{{\varepsilon_{Y} (U)}}{{\eta_{Y} (U)}}t}} } \right)\frac{\sigma }{{E_{Y} (U)}} + \frac{{\sigma - \sigma_{s} }}{{\eta_{N} (U,t)}}t,t > t_{s} \hfill \\ \end{aligned} \right. $$

$\varepsilon (t)$ is the principal equation of the Nishihara model considering the weakening effect of pre-peak unloading damage.

### Creep model validation

**Figure 18:**
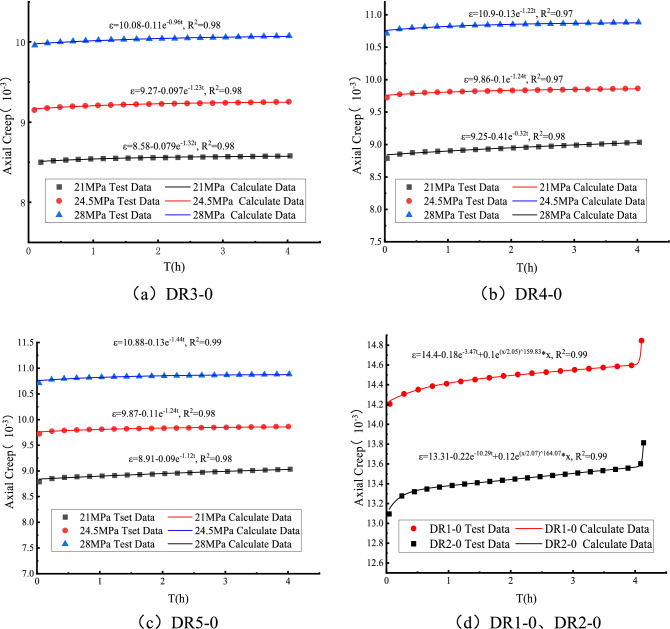
Comparison between experimental data of white sandstone and theoretical curve.

Based on the uniaxial creep test data of white sandstone with pre-peak unloading damage, using Matlab software and using the improved Leweinberg-Marquardt algorithm to analyze and simulate the model parameters in the Nishihara model considering the pre-peak unloading damage, and the simulation results are shown in Tables 5, 6, and 7. In order to more intuitively compare the changes between the theoretical curve and the actual creep test curve, the test data of the pre-peak unloading damage $\sigma_{\mu l}$ = 70%, 80% and 90% are selected for analysis, as shown in Fig. 18.

**Table 5:** Pre-peak unloading damage model identification parameters under non-yield stress conditions (same damage degree).

*σ*/MPa	*σ*_*ul*_/%	*E*_*H*_*(U)*/MPa	*E*_*Y*_*(U)*/MPa	*η*_*Y*_*(U)*/MPa
30	50	2.816	118.8	96.69
35		2.962	147.46	132.1

**Table 6:** Identification parameters of pre-peak unloading damage model under non-yield stress conditions.

*σ*/MPa	*σ*_*ul*_/%	*E*_*H*_*(U)*/MPa	*E*_*Y*_*(U)*/MPa	*η*_*Y*_*(U)*/MPa
21	70	2.47	298	227.6
	80	2.416	264.65	208.33
	90	2.38	233.96	200.63
24.5	70	2.67	251.8	205.1
	80	2.51	226.85	183.23
	90	2.5	226.84	183.12
28	70	2.808	244.98	253.64
	80	2.61	221.68	166.43
	90	2.6	221.43	153.43

**Table 7:** Identification parameters of pre-peak unloading damage model under yield stress conditions.

*σ*/MPa	*σ*_*ul*_*/*%	*E*_*H*_*(U)*/MPa	*E*_*Y*_*(U)*/MPa	*η*_*Y*_*(U)*/MPa	*η*_*N*_*(U)*/MPa	*λ*	*θ*
50	50	3.517	278.3	80.24	148.1	2.055	159.8
42.5	60	3.243	198.2	19.5	102.9	2.072	163.11

Tables 5, 6, and 7 show the data tables of the identification parameters of the Nishihara model considering the effect of pre-peak unloading damage, from which it can be seen that the elastic modulus and viscosity coefficients in the Nishihara model decrease with the increase of the degree of pre-peak unloading damage. This is because the rock undergoes pre-peak unloading damage and weakening, and the internal structure is damaged, resulting in the continuous weakening of mechanical properties. Table 5 shows that at $\sigma_{\mu l} = {5}0\%$, with the increase of creep stress, the elastic modulus and viscosity coefficient of the pre-peak unloading damage creep model both increase, and the coefficient increases by a similar amount. Table 6 shows that the creep model parameters of white sandstone with the same degree of damage increased with the creep stress level at $\sigma_{\mu l} = 70\% ,\;80\% ,\;90\%$; however, the elastic modulus and viscosity parameters gradually decreased with the increase of the peak front unloading damage at the same creep stress level.

Figure 18a–c shows the comparison of experimental data and theoretical curves for DR3-0, DR4-0 and DR5-0 under non-yield stress conditions, while Fig. 18d shows the comparison between the experimental data and the theoretical curve of the unloading damage degree before the peak under the condition of yield stress (including the accelerated creep stage). Figure 18 shows the theoretical curve of the Nishihara creep model of white sandstone considering pre-peak unloading damage, whether the white sandstone is in the yield stress state or not, it can better describe the uniaxial creep process of the white sandstone with pre-peak unloading damage. Taking Fig. 18d as an example, when the peak front unloading damaged white sandstone is in the yield stress state, although the accelerated creep curve is difficult to describe, the Nishihara creep model of white sandstone considering pre-peak unloading damage can still accurately describe its change process. And the experimental curve is highly consistent with the theoretical curve, the linear correlation regression coefficient R^2^ ≥ 0.99, which shows that the creep model can better describe the whole process of creep of peak front unloading damage.

Figure 18 shows: (1) The theoretical simulation of the Nishihara creep model considering the pre-peak unloading damage of white sandstone has a very high degree of fit, which shows that the creep model can describe the stability of the specimen when it is in a state of non-yield stress. In the creep stage, the strain changes from nonlinear growth to linear growth viscoelasticity, which can also accurately describe the viscoplastic features of the unstable creep stage when the specimen is in a state of yield stress, and the specimen undergoes an accelerated creep stage to produce plastic deformation, and compared with previous studies, it can be found that the Nishihara model considering the pre-peak unloading damage effect can more accurately describe the characteristics of the accelerated creep stage when the rock is in the state of yield stress. (2) Fig. 18d and Table 7 show that the model is based on the aging damage effect, thus introducing a two-parameter Weibull distribution function, the parameters $\theta$, $\lambda$ in the function have an important effect on the curve description of the accelerated creep stage of the rock under the yield stress state, and the changes of the two are inversely proportional. When the damage parameter $\theta$ is larger and $\lambda$ is smaller, the accelerated creep stage is more obvious, the degree of curve change is larger, and the axial accelerated creep strain rate of the specimen is larger, so that the internal structure of the rock specimen changes from crack quantity to qualitative change. That is, from the increase in the number of cracks to the formation of macroscopic crack bands, the shorter the specimen is required for aging. When $\lambda$ is larger, $\theta$ is smaller, indicating that the curve change of rock accelerated creep stage under the yield stress state has obvious time effect, that is, with the increase of creep time, the axial creep deformation rate of rock increases nonlinearly and slowly with time showing a non-linear trend.

To sum up, the mechanism of action is that under the condition of yield load, the crystals inside the rock rub and slip with each other, and a new stress structure is continuously formed to resist the external load. With the gradual accumulation of microscopic defects, the effective area corresponding to the stress gradually decreases, resulting in local stress concentration, the surrounding micro-cracks rapidly expand into macro-sections, and the rock becomes unstable and its strain surges. Considering the pre-peak unloading damage effect, the Nishihara model can better describe the creep deformation of the surrounding rock in the deep well roadway during the re-mining process, and provides a certain reference value.

## Conclusion

In this paper, the macroscopic mechanical properties and the fine structure of the damage were analyzed by XRF, XRD, SEM and uniaxial creep tests. The effects of the pre-peak unloading damage and the fine structure on the long-term strength and damage properties of the white sandstone were investigated and their mechanisms. The conclusions are as follows.

1. The results of creep tests on white sandstone with pre-peak unloading damage show that all stages of sandstone creep are affected by the effect of pre-peak unloading damage significantly. Specifically, as the degree of pre-peak unloading damage increases, the specimen transitions more quickly from the decaying creep stage to the stable creep stage, the corresponding creep deformation volume and rate are significantly increased, while the stress threshold into the accelerated creep stage gradually decreases, the time experienced from stable to accelerated creep is shorter.
2. Under long-term loading, the macroscopic rupture pattern of white sandstone evolves from "splitting damage"—"single bevel shear damage"—"mixed splitting-shear damage" as the degree of pre-peak unloading damage increases, the mechanism of damage lies in the development of microfractures within the rock during pre-peak unloading damage and the time-dependent damage that promotes the penetration of microfractures.
3. A comparison of the creep test data of the pre-peak unloading damaged white sandstone with the theoretical curve of the Nishihara model considering pre-peak damage shows that the model not only reflects the viscoelastic deformation behavior of the pre-peak unloading damaged sandstone during the decaying and stabilizing creep phases, but also describes the viscoplastic characteristics of its strain evolution from non-linear growth to plastic flow during the accelerated creep phase.

## Data Availability

The datasets generated and analyzed during the current study are not publicly available as the datasets are from the team and are only available for this study, but datasets are available from the corresponding author on reasonable request.
